# Evaluation of the undergraduate family medicine programme of Faculty of Medicine, University of Kelaniya: quantitative and qualitative student feedback

**DOI:** 10.1186/s12909-019-1882-6

**Published:** 2019-12-02

**Authors:** D. P. Perera, S. S. Withana, K. Mendis, D. V. T. Kasunjith, W. T. S. Jayathilaka, S. Wickramasuriya

**Affiliations:** Department of Family Medicine, Faculty of Medicine, University of Kelaniya, PO Box 6, Thalagolla Road, Ragama, Sri Lanka

## Abstract

**Background:**

Worldwide there is an increasing emphasis on the importance of primary care. The ministry of health Sri Lanka issued a directive in 2016 that training of doctors in primary care should be strengthened.

Medical students of the Faculty of Medicine, University of Kelaniya follow a 1 month long clinical appointment in family medicine in their fourth year of study.

**Methods:**

Feedback is taken from students on completion of the appointment. Half the students from each group complete a pre tested structured feedback questionnaire that consists of answers to questions based on a likert scale with a space for free comments. The other half provide qualitative feedback.

In this evaluation data were gathered from 185 (98%) students from all eight clinical groups throughout the year 2016.

Quantitative data were analysed using SPSS version 22. Inductive thematic analysis was used to analyse the qualitative data from the Round Robin activity and free comments from the questionnaire.

**Results:**

The qualitative feedback provided a richer indepth overview of student ideas on the appointment compared to the quantitative data.

In reflection of a desire for learning to be of relevance students wanted clinically oriented teaching focused on management. They preferred active teaching learning methods such as the opportunity to conduct consultations and receive immediate feedback. Students had a high regard for the teaching sessions by general practitioners at their clinics.

The appointment had created an interest in the discipline of family medicine which could have an impact on future choice of career. There were indications to suggest that student attitudes towards patients may have evolved to be more patient centred.

Students appreciated the inclusive and low stress ambience of the learning environment.

**Conclusions and recommendations:**

Regular evaluation of teaching programmes helps maintain accountability of faculty and paves the way for more student centred teaching through the incorporation of students’ views in devising teaching methods. This evaluation found that qualitative feedback provided more descriptive material to reflect on and therefore improve teaching on the programme. It is recommended that more use should be made of qualitative methodologies in programme evaluations.

## Background

Family medicine is the discipline that is geared towards provision of high quality health care based on the principles of first contact, comprehensive, coordinated, personalised care and preventive and health promotive activities. It is the only specialty that provides care to the whole family. Globally, it is now widely recognised that a disease oriented approach is becoming increasingly dysfunctional and that it must be replaced by a focus on people and populations with their unique combinations of illnesses rather than specific diseases [1]. With the increasing emphasis on the importance of primary care the ministry of health Sri Lanka issued a directive in 2016 that the training of doctors in primary care should be strengthened [2].

Medical students of the Faculty of Medicine, University of Kelaniya follow a 1 month long clinical appointment in family medicine at the University Family Practice Centre in their fourth year of study.

Teaching is conducted through a variety of teaching and learning methods. Students engage in traditional patient clerking, observe day to day activities of the clinic, manage the medical records system and also learn clinical examination techniques. They are given the opportunity to conduct consultations themselves and are given one to one feedback on their consultations. Small group discussions are conducted based on common reasons for encounter in a family practice.

Students visit a general practice (GP) clinic for three teaching sessions and have one visit to the outpatient department of the Colombo North teaching hospital.

At the end of the appointment students participate in a seminar and debate. They present data on the spectrum of morbidity encountered during their visits to the GP. They also formulate a proposed layout for an ideal GP clinic.

The assessment at the end of the appointment consists of two structured essay questions based on clinical cases and principles of family medicine.

At the end of the appointment quantitative and qualitative feedback is collected from students.

Many evaluations of undergraduate family medicine clerkships have been conducted in other countries. Most of them use a structured self-administered questionnaire to gather data [3–5].

A questionnaire based student feedback in an Austrian undergraduate setting found that students viewed the family medicine clerkship as an essential aspect of their education and were highly satisfied with the appointment [4].

In a questionnaire based evaluation by students at the King Saud University, College of Medicine, Saudi Arabia students appreciated learning about the caring and communication aspects of patient care. The study showed that practical procedural skills are desirable features of a preceptorship programme and that an emphasis on doing versus observing is preferred by students [5].

Written evaluations of the fourth-year medical student attachment in general practice were obtained from 75 medical students at the University of Dundee to determine the strengths and weaknesses of the teaching programme. Interviews were also conducted with students and their tutors and a focus group was arranged at the conclusion of the attachment. The overall evaluation by the students was positive. Students liked the opportunity for the hands-on practice of medicine and the collegial reception from their tutors. Major criticisms related to the lack of adequate opportunities for some students to see patients on their own and to learn practical procedures [6].

Publications on undergraduate student feedback on teaching and learning in Sri Lanka are scarce. In an evaluation of the teaching approaches used in the biochemistry course for second year medical students of the Rajarata University two questions were administered to students who completed the second MBBS Objective Structured Practical Examination (OSPE) in Biochemistry. The first question was a fixed response question whilst the second was a free response question. Lectures were the most popular method of teaching while other preferred methods were student staff interaction, panel discussion and the least preferred method was seminar [7].

A previous survey was done in the same setting as this study in 2014 using a pretested self-administered structured questionnaire with space at the end for open ended comments. The questionnaire was administered to six consecutive clinical groups at the end of the 1 month clinical appointment. This survey showed that direct observation of student consultations and feedback from teachers was the most popular teaching method among students while need to strengthen hands on learning methods such procedural skills and clinical examination techniques was emphasised [8].

Since 2016 student feedback has routinely been obtained using the same structured questionnaire that was used in the previous evaluation in this setting to gather data from half the students in each group. A qualitative round robin data gathering method is used to gather data from the other half of the students in each group.

The aim of this study was to gain a comprehensive view of student perceptions of the family medicine appointment.

## Methods

Data were gathered from 185 (98%) students from all eight clinical groups throughout the year 2016. Feedback was taken at the end of the clinical appointment from each group.

### Method I – questionnaire

During the feedback activity each group is divided into two according to the register. Half of the students fill in the pre-tested structured feedback questionnaire that consists of questions with responses based on a Likert scale with a space for free comments as well. Additional file 1 shows the structured questionnaire.

### Method II – round Robin activity

The other half of each group provides qualitative feedback using a Round Robin method of brainstorming. During this activity each student is asked to write a free non prompted comment regarding the appointment and pass his feedback around the table to the next student who can either add another comment or indicate agreement (with a tick) or disagreement (with a cross) to the idea expressed by the initial student. This addition of comments and ideas continues till no more new comments are added and the point of saturation is reached. The time taken for this process is approximately 45 min for each group. Variations of this method have been used in many settings to get programme evaluation feedback and suggestions for improvement from students as it provides semi quantitative data in a way that actively engages students while allowing equal opportunity for all students to share their views [9–13].

### Data analysis

Quantitative data analysis was done using SPSS version 22. Qualitative data from the round table method and qualitative data from the free comments in the questionnaire were analysed separately. Thematic analysis was used to identify, analyse and report patterns within the qualitative data [14]. An inductive data driven method was used where three researchers read and re read the data and coded the data independently. The codes were categorised into themes that were further refined and validated by extensive discussion among the researchers. The team of researchers had varied medical education backgrounds and were at different levels in their careers. The quantitative and qualitative data were scrutinised for convergence, complementarity or dissonance [15]. The numbers of students agreeing to a specific comment or disagreeing to a specific comment in the round robin group were taken into account in developing themes. However, views that were not supported by a large number of people but were considered important and reflective of diverse student experience were given thoughtful attention [14].

## Results

### Quantitative results from the questionnaire

Students were given the option to agree or disagree to statements evaluating the usefulness of the various aspects of the appointment on a Likert scale. Students had provided agree and strongly agree answers to most of the statements.

Students stated that they had a clear idea of the learning outcomes for the appointment. They stated that they had gained an adequate understanding of the basic concepts of family medicine and organisational aspects of a family practice. They were satisfied with the opportunity they got to improve communication skills, history taking, problem solving and presentation skills. However only 47% of students had agreed to the fact that they had got the opportunity to develop skills in clinical examination. Student responses to the question stating that they had acquired a basic knowledge of common diseases were ambivalent with this question not being answered by 54%. The teaching methods they were most appreciative of were learning from patients followed by the debate and performing consultations under observation and receiving feedback. Table 1 describes the quantitative findings.

**Table 1:** Quantitative results of the questionnaire

	Disagree	Not sure	Agree	No response
1.I had a clear idea of the learning outcomes for this appointment				
	2%	2%	96%	0%
2.I gained an adequate understanding of the following concepts of family medicine				
a) First contact care	1%	2%	96%	1%
b) Personalised care	1%	2%	96%	1%
c) Prevention and health promotion	1%	4%	94%	1%
d) Comprehensive care	1%	4%	93%	2%
e) Coordination of care	1%	7%	90%	2%
f) Continuity of care	1%	3%	93%	3%
3.I acquired a basic knowledge of common illnesses seen in family practice and their management				
	0%	1%	45%	54%
4. During this appointment I had the opportunity to develop the following skills				
a) Communication	1%	5%	94%	0%
b) History taking	2%	3%	94%	1%
c) Clinical examination	15%	38%	47%	0%
d) Problem solving/analytic	3%	24%	73%	0%
e) Presentation (histories/seminars/debate)	2%	5%	92%	1%
5.I gained an understanding of the organisational aspects of a general practice: office layout, appointment system etc.				
	3%	3%	72%	22%
6.During the appointment the following teaching methods facilitated my learning				
a) Small group discussions	16%	21%	60%	3%
b) Learning from patients	1%	3%	93%	3%
c) Direct observation and feedback from teachers	2%	5%	89%	4%
d) Seminar	1%	20%	77%	2%
e) Debate	2%	8%	89%	1%
f) Preparation and presentation of the group topic	5%	18%	73%	4%

### Qualitative results of the free comments from the questionnaire

Students were given space in the questionnaire to write free comments on what was good and what was in need of improvement regarding teaching and learning during the appointment. 80% of students had written at least one comment. It was noted that the themes that arose were similar to that of the qualitative feedback only group.

The number of themes were lesser than from the round table method. Two main ideas were stressed regarding the need for further emphasis on clinically oriented teaching focused on primary care management rather than hospital management and inadequate availability of facilities such as space for patient examination and adequate equipment for patient examination.

### Qualitative results from the round table activity

#### Teaching methods

Students rated being able to conduct consultations independently and then receiving one to one feedback highly. “Giving opportunity to consult a patient in front of a doctor is good thing for us to improve our consultation skills.”

The majority disliked didactic lecture style teaching and preferred case based interactive discussions. They mentioned that they would have appreciated more time for discussion of the patients they had seen in the clinic.

The end of appointment debate was also one of the learning opportunities that was valued by most students. Students recognised that it gave an opportunity to engage “students who did not usually participate.” However, many students requested that a new topic for debate should be given to each group without repeating the same topic for each group.

Interestingly, although many students complained about the distance they had to travel to visit the GP practices “tendency to get RTA (road traffic accident)” a larger number appreciated the experience claiming that it gave them the chance to observe an “authentic GP setting” and see the “GP approach to patients.” Students described the GP trainers as “friendly” and “enthusiastic in their teaching.”

Students also enjoyed the home visits and requested more exposure to home visits.

There was the general view that there should be more opportunity for practical hands on work at the clinic. Students said they would have liked more exposure to procedures such as wound care, nebulisation etc. They also mentioned that they had not had enough opportunities to practice examination skills or observe teacher demonstration of examination techniques.

#### Impact on knowledge, skills, attitudes and future practice

Students believed that common topics likely to be encountered during general practice were covered during the appointment and their prior learning was refreshed. It was stated that the topics were “appropriate”, “pitched at the correct level” and “covered all common diseases”.

Students requested that more emphasis should be given to teaching the management aspect.

Students suggested that the clinic should have a pharmacy stocked with the common medications used in a family practice to have an opportunity for learning about medicines.

Having the opportunity to register patients, retrieve medical records and get first-hand experience as a practice manager was highly appreciated. “Your method helped us to do it ourselves and we will remember the idea for a life time.”

“Doing a lecture on medical records would have been boring.”

The appointment seemed to have had a positive impact on student communication skills. Students said that they were able to learn how to build a good rapport with the patient and how to improve their communication skills.

It was a surprising finding that students revealed that they were hesitant to take time from the patients’ visit for learning purposes. “Cannot trouble patients in this setting as we do in the hospital.”

They were sensitive to the fact that patients were kept longer when students were in the clinic.

“Patients are kept waiting for a long time (when teaching is being carried out).”

The appointment had kindled an interest regarding the field of family medicine in many. It was stated that the experience has given them a clear view of how to establish their own practice in future. One student said “your effort to make family medicine a stream that many students will pursue was not in vain. I feel that it’s a good stream for a doctor to have less stress and much satisfaction.”

“Seeing how the patients benefit from the consultations, counselling made the field seem much more interesting.”

#### Staff and learning environment

The fact that the learning environment was student friendly and free of stress was highly appreciated. “Staff in the family medicine department has this different vibe”.

“Feels like a family-well, may be that suits its’ name”.

“Very enthusiastic and not stressful which facilitates learning.”

Students valued the authenticity of the setting both at the university clinic and the GP clinics. It was said that it helped them in “getting an idea about how general practice is different from the clinical experience of hospital only to which we’ve been exposed so far.”

During the appointment students learn various skills from the practice nurse, lab technician and administration staff regarding patient care, lab investigations and day to day clinic management. Students appreciated the support given to them by all categories of staff.

“All staff; professors, doctors, demonstrators, clerk and lab technician put great effort and conducted the appointment in a professional way”.

Students complained that there was inadequate space for them to take histories and examine patients in the clinic.

## Discussion

A majority of studies evaluating undergraduate family medicine clerkships use a quantitative methodology of students filling a questionnaire based on a likert scale [3–5]. When formulated in a systematic manner this method of evaluation has been found to be valid and useful [16]. However, the value of qualitative feedback for programme evaluation is being increasingly reinforced both in health education and wider fields of teaching [9, 17, 18]. It was thought worthwhile to reflect on the differences in the two types of data collected. In this evaluation student free comments from the round table discussion appeared to be well thought out and specific details and examples had been given explaining what worked and what did not. The qualitative feedback provided a richer and in-depth overview of student ideas on the appointment that are more useful with regard to implementation of future changes in teaching and learning. The number of students who had agreed with or disagreed with a specific statement in the round robin group helped give an idea of how widely and strongly a specific opinion was held within the group. The qualitative findings also facilitated the revelation of certain unexpected and intangible findings related to attitudes and behaviour that could not have been gauged from a structured questionnaire with pre determined questions.

Data from the questionnaire were mostly complementary and converged with the qualitative findings. However, some limitations of using a structured questionnaire in programme evaluation are highlighted. The majority of statements in the questionnaire received agree and strongly agree answers which questions the quality of the data. Furthermore, there was a 54% non response to the question on whether students acquired a basic knowledge of common illnesses seen in family practice and their management. This illustrates the ambiguity and difficulty in interpreting numbers with regard to direction for improvement when there is significant non response to an item and the reason for non response is not known.

In this study students emphasised that teaching should be more clinically oriented with more opportunity for practical hands on learning in a reflection of their adult learner identity. In a reflection of a desire for learning to be of relevance to assessment and practice they mentioned that they would have valued more exposure to conducting procedures, patient examination and clinically oriented teaching focused on management. These findings closely follow findings of similar studies done in undergraduate family medicine clinical teaching settings which emphasise that student prefer active and hands on learning styles [5, 6, 8].

The fact that students felt that they could not “trouble” the patients at the family practice in taking histories, examining them and therefore taking extra time from the patients’ visit was an interesting finding and a subtle indicator that perhaps students had experienced an understanding of the difference in the clinical environment at a family practice where patients have more autonomy in comparison to in ward patients. It could be hypothesised that exposure to a first contact ambulatory primary care environment had an impact on student attitudes in line with patient centred care. Some previous studies in general practice teaching settings have found that student participation during consultations increases consultation time and raises issues of confidentiality. Despite this finding studies also show that patients are mostly happy for students to be present during consultations with their GP. While students in this study may have felt they were wasting the patient’s time and their presence did not add anything to the consultation previous studies show that patients felt that they benefitted from the presence of a student as they were able to know more details about the illness from students and students helped in revealing details to the doctor [19–23].

In our setting students take the patient’s history before coming into the consultation room, and then present the history to the teaching doctor in front of the patient. This method has been shown to be more time efficient than the student presenting histories to the teacher separately but still results in an increase in the consultation time [24]. Students also perform at least one complete consultation from start to finish under the observation of the supervisor with supervisor intervention when and where appropriate. This method increases the time for teaching even more.

Despite the increasing recognition of the need to strengthen primary care family medicine continues to commonly be a default career option [25]. Recent Ministry of health plans for primary health care reforms in Sri Lanka acknowledge that the principles of family medicine need to be integrated into health training so that attitudes and practices of primary level personnel are adapted to this approach [26]. Studies indicate that exposure to an undergraduate family medicine clinical appointment has a positive impact on student attitudes towards family medicine [3]. In this current evaluation it was evident that student attitudes towards family practice had improved at the end of the appointment. In order to motivate more students to actively pursue a career in family medicine it is imperative that undergraduate teaching and training in family medicine should be carefully planned out to highlight the positive aspects of a career in family medicine. The programme should be perceived as a valuable part of undergraduate medical education.

Students highlighted the importance of a conducive learning environment in facilitation of learning. While students were unhappy about the lack of adequate space and equipment necessary for patient examination the attitudes of the teaching and support staff involved in the family medicine programme seem to have had a positive impact on student learning. The structure of the family medicine programme facilitates positive interaction with a team of individuals from different professions and staff categories. Students highlighted the support given to them by the whole team as an important motivator of learning.

The findings of this evaluation led to changes being made to the programme. The small group discussions were planned to be more clinically oriented and the number of sessions were reduced to allow for more time for interaction with and learning from patients facilitated by teachers within the consultation room. Students were allocated more space to talk to patients and examine them and new topics were introduced for the debate.

### Limitations

Half of the students in each clinical group were allocated to give feedback using the questionnaire and the other half participated in the Round Robin qualitative feedback activity. In order to allow for more robust comparison of qualitative and quantitative feedback it may have been better to request the whole student group to fill in the questionnaire followed by recruitment of a sample of the same group to participate in the qualitative feedback activity.

## Conclusions and recommendations

Regular evaluation of teaching programmes helps maintain accountability of faculty and paves the way for more student centred teaching through the incorporation of students’ views in devising teaching methods. This evaluation found that qualitative feedback provided more descriptive material to reflect on and therefore improve teaching on the programme. It is recommended that more use should be made of qualitative methodologies in programme evaluations.

## Supplementary information

**Additional file 1.** Questionnaire for evaluation of the teaching and learning on the undergraduate family medicine appointment at the Faculty of Medicine, University of Kelaniya.

## Data Availability

The original data are stored at the Department of Family Medicine and not freely available for sharing as there is identifiable information regarding trainers.

## Abbreviations

GP: General practice
